# Prospective prediction of substances use among a cohort of Tunisian adolescents

**DOI:** 10.1093/eurpub/ckac131.466

**Published:** 2022-10-25

**Authors:** N Zammit, A Amara, R Ghammam, S Ben Fredj, A Maatouk, M Ouertani, W Ben Belgacem, S Boujebha, J Maatoug, H Ghannem

**Affiliations:** 1 Epidemiology, University Hospital Farhat Hached, Sousse, Tunisia; 2 Faculty of Medicine of Sousse, University of Sousse, Sousse, Tunisia; 3 LR19SP03, University Hospital Farhat Hached, Sousse, Tunisia; 4 Department of Family and Community Medicine, Faculty of Medicine of Sousse, University of Sousse, Sousse, Tunisia; 5 LR12ES03, Faculty of Medicine of Sousse, University of Sousse, Sousse, Tunisia

## Abstract

**Background:**

The risk of substances use increases during adolescence. In Sousse (Tunisia), an upward trend of these risk behaviors has been observed during the last years among young adolescents. Among older adolescents, the trend of their use is unknown.

**Objectives:**

To determine the incidences and the most influencing factors on substances use among high school students in Sousse between 2018 and 2019.

**Methods:**

A prospective longitudinal study was conducted among a cohort of high school students from Sousse. The same pre-tested questionnaire served to collect data in 2018 and 2019 from the same participants in their classes and in the presence of pre-trained investigators.

**Results:**

A total of 404 high school students have participated in the study. Their median age was of 17 (IIQ: 15.8-17.6) years. Girls represented 66.8% of participants. The incidence rates of lifetime tobacco use, alcohol consumption, lifetime inhalants use and lifetime illicit substances use between 2018 and 2019 were 13%, 3.5%, 1.8% and 2 .9% respectively. Lifetime tobacco use was the main predictor of inhalants experimentation. This latter was the main predictor of becoming a user of e-cigarettes while alcohol consumption was the most influencing factor on cannabis experimentation among high school students. On the other hand, illicit substances use among friends predicted e-cigarette use, alcohol consumption, and cannabis experimentation among participants.

**Conclusions:**

The existing prevention programs aiming at reducing tobacco use and substances use in the schools of Tunisia should be reinforced and integrate a comprehensive and multi-sectoral prevention program. The implementation of a national observatory of substances use would ensure the continuous improvement of this program.

**Key messages:**

• There is an upward trend on using substances among the adolescents of Sousse, Tunisia.

• Tobacco experimentation and alcohol consumption are the gateway to later substances experimentation among the adolescents of Sousse, Tunisia.

